# Supplementary material for: Remote control of microtubule plus-end dynamics and function from the minus-end
Source: eLife. 2019 Sep 6;8:e48627. doi: 10.7554/eLife.48627 (PMC6754230; doi:10.7554/eLife.48627)
Supplement: Supplementary file 2. [file elife-48627-supp2.docx]

Supplementary File 2.

Strains used in this study.

**­**

| **Yeast (yYB)**  **Strain number** | **Mating**  **type** | **Genotype** | **Background** |
| --- | --- | --- | --- |
| 14590 | a | *Kip3-3xsfGFP:KanMX Spc42-mCherry:NatMX*  *ura3-52 his3Δ200 leu2 lys2-801 trp1Δ63 Ade2+* | S288C |
| 15100 | a | *Kip2-3xsfGFP:KanMX Spc42-mCherry:NatMX*  *ura3-52 his3Δ200 leu2 lys2-801 trp1Δ63 Ade2+* | S288C |
| 15102 | a | *kip2∆::Kip2-S63A-3xsfGFP:KanMX Spc42-mCherry:NatMX*  *ura3-52 his3Δ200 leu2 lys2-801 trp1Δ63 Ade2+* | S288C |
| 15692 | a | *Kip2-3xsfGFP:KanMX Spc42-mCherry:NatMX bfa1::TRP*  *ura3-52 his3Δ200 leu2 lys2-801 trp1Δ63 Ade2+* | S288C |
| 15693 | a | *Kip2-3xsfGFP:KanMX Spc42-mCherry:NatMX bub2::hphNT1*  *ura3-52 his3Δ200 leu2 lys2-801 trp1Δ63 Ade2+* | S288C |
| 15794 | a | *Kip2-3xsfGFP:KanMX Spc42-mCherry:NatMX*  *bfa1::TRP bub2::hphNT1*  *ura3-52 his3Δ200 leu2 lys2-801 trp1Δ63 Ade2+* | S288C |
| 10414 | a | *Kip3-3xsfGFP:KanMX4 Spc72-GFP:His3MX*  *ura3-52 his3Δ200 leu2 lys2-801 trp1Δ63 Ade2+* | S288C |
| 9806 | a | *Kip2-3xsfGFP:KanMX4 Spc72-GFP:His3MX*  *ura3-52 his3Δ200 leu2 lys2-801 trp1Δ63 Ade2+* | S288C |
| 10676 | a | *kip2∆::Kip2-S63A-3xsfGFP:KanMX4 Spc72-GFP:His3MX*  *ura3-52 his3Δ200 leu2 lys2-801 trp1Δ63 Ade2+* | S288C |
| 15105 | a | *kip2∆::Kip2-G374A-3xsfGFP:KanMX Spc42-mCherry:NatMX*  *ura3-52 his3Δ200 leu2 lys2-801 trp1Δ63 Ade2+* | S288C |
| 15176 | a | *kip2∆::Kip2-G374A-3xsfGFP:KanMX Spc72-mCherry:NatMX*  *ura3-52 his3Δ200 leu2 lys2-801 trp1Δ63 Ade2+* | S288C |
| 15107 | a | *kip2∆::Kip2-S63A-G374A-3xsfGFP:KanMX*  *Spc42-mCherry:NatMX*  *ura3-52 his3Δ200 leu2 lys2-801 trp1Δ63 Ade2+* | S288C |
| 15777 | a | *kip2∆::Kip2-S63A-G374A-3xsfGFP:KanMX*  *Spc42-mCherry:NatMX bfa1::TRP*  *ura3-52 his3Δ200 leu2 lys2-801 trp1Δ63 Ade2+* | S288C |
| 15775 | a | *kip2∆::Kip2-S63A-G374A-3xsfGFP:KanMX*  *Spc42-mCherry:NatMX bub2::KanMX*  *ura3-52 his3Δ200 leu2 lys2-801 trp1Δ63 Ade2+* | S288C |
| 15778 | a | *kip2∆::Kip2-S63A-G374A-3xsfGFP:KanMX*  *Spc42-mCherry:NatMX bfa1::hphNT1 bub2::KanMX*  *ura3-52 his3Δ200 leu2 lys2-801 trp1Δ63 Ade2+* | S288C |
| 15187 | diploid | *Kip2-G374A-3xsfGFP:KanMX/Kip2-mCherry:hphNT1*  *ura3-52 his3Δ200 leu2 lys2-801 trp1Δ63 Ade2+* | S288C |
| 15336 | diploid | *Kip2-S63A-G374A-3xsfGFP:KanMX/Kip2-mCherry:hphNT1*  *ura3-52 his3Δ200 leu2 lys2-801 trp1Δ63 Ade2+* | S288C |
| 14269 | a | *kip2∆::Kip2-6HA:NatMX*  *ura3-52 his3Δ200 leu2 lys2-801 trp1Δ63 Ade2+* | S288C |
| 14405 | a | *kip2∆::Kip2-S63A-6HA::NatMX*  *ura3-52 his3Δ200 leu2 lys2-801 trp1Δ63 Ade2+* | S288C |
| 11068 | alpha | *Bik1-3xGFP:hyg Spc72-GFP:His3MX*  *ura3-52 his3Δ200 leu2 lys2-801 trp1Δ63 Ade2+* | S288C |
| 11069 | a | *Bik1-3xGFP:hyg Spc72-GFP:His3MX*  *ura3-52 his3Δ200 leu2 lys2-801 trp1Δ63 Ade2+* | S288C |
| 11365 | a | *Bik1-3xGFP:hyg Spc72-GFP:His3MX kip2∆::Kip2-S63A:TRP*  *ura3-52 his3Δ200 leu2 lys2-801 trp1Δ63 Ade2+* | S288C |
| 12564 | alpha | *Bik1-3xGFP:hyg Spc72-GFP:His3MX kip2∆::Kip2-wt:TRP*  *ura3-52 his3Δ200 leu2 lys2-801 trp1Δ63 Ade2+* | S288C |
| 14218 | a | *Bik1-3xGFP:hyg Spc72-GFP:His3MX bfa1::TRP*  *ura3-52 his3Δ200 leu2 lys2-801 trp1Δ63 Ade2+* | S288C |
| 14219 | a | *Bik1-3xGFP:hyg Spc72-GFP:His3MX bub2::KanMX*  *ura3-52 his3Δ200 leu2 lys2-801 trp1Δ63 Ade2+* | S288C |
| 14217 | a | *Bik1-3xGFP:hyg Spc72-GFP:His3MX bfa1::TRP bub2::KanMX*  *ura3-52 his3Δ200 leu2 lys2-801 trp1Δ63 Ade2+* | S288C |
| 15541 | alpha | *Dyn1-mNeonGreen:NAT*  *Spc42-mCherry:NAT Bik1-3xmCherry:HIS*  *ura3-52 his3Δ200 leu2 lys2-801 trp1Δ63 Ade2+* | S288C |
| 15864 | alpha | *Dyn1-mNeonGreen:NAT kip2::hphNT1*  *Spc42-mCherry:NAT Bik1-3xmCherry:HIS*  *ura3-52 his3Δ200 leu2 lys2-801 trp1Δ63 Ade2+* | S288C |
| 15865 | alpha | *Dyn1-mNeonGreen:NAT kip2∆::Kip2-G374A:TRP*  *Spc42-mCherry:NAT Bik1-3xmCherry:HIS*  *ura3-52 his3Δ200 leu2 lys2-801 trp1Δ63 Ade2+* | S288C |
| 15542 | alpha | *Dyn1-mNeonGreen:NAT kip2∆::Kip2-S63A:TRP*  *Spc42-mCherry:NAT Bik1-3xmCherry:HIS*  *ura3-52 his3Δ200 leu2 lys2-801 trp1Δ63 Ade2+* | S288C |
| 15791 | alpha | *Dyn1-mNeonGreen:NAT bfa1::TRP*  *Spc42-mCherry:NAT Bik1-3xmCherry:HIS*  *ura3-52 his3Δ200 leu2 lys2-801 trp1Δ63 Ade2+* | S288C |
| 15792 | alpha | *Dyn1-mNeonGreen:NAT bub2::KanMX*  *Spc42-mCherry:NAT Bik1-3xmCherry:HIS*  *ura3-52 his3Δ200 leu2 lys2-801 trp1Δ63 Ade2+* | S288C |
| 15793 | alpha | *Dyn1-mNeonGreen:NAT bfa1::TRP bub2::hphNT1*  *Spc42-mCherry:NAT Bik1-3xmCherry:HIS*  *ura3-52 his3Δ200 leu2 lys2-801 trp1Δ63 Ade2+* | S288C |
| 9738 | a | *Spc72-GFP:His3MX*  *ura3-52 his3Δ200 leu2 lys2-801 trp1Δ63 Ade2+* | S288C |
| 11522 | a | *Spc72-GFP:His3MX dyn1::HYG*  *ura3-52 his3Δ200 leu2 lys2-801 trp1Δ63 ade1-101* | S288C |
| 11878 | a | *Spc72-GFP:His3MX Kip3-3xsfGFP:KanMX dyn1::HYG*  *ura3-52 his3Δ200 leu2 lys2-801 trp1Δ63 Ade2+* | S288C |
| 11076 | a | *Spc72-GFP:His3MX bim1::hphNT1*  *ura3-52 his3Δ200 leu2 lys2-801 trp1Δ63 Ade2+* | S288C |
| 11078 | a | *Spc72-GFP:His3MX bim1::hphNT1 Bik1-3xGFP:HYG*  *ura3-52 his3Δ200 leu2 lys2-801 trp1Δ63 Ade2+* | S288C |
| 11048 | a | *Spc72-GFP:His3MX bim1::hphNT1 Kip2-3xsfGFP:KanMX4*  *ura3-52 his3Δ200 leu2 lys2-801 trp1Δ63 Ade2+* | S288C |
