# Supplementary material for: Remote control of microtubule plus-end dynamics and function from the minus-end
Source: eLife. 2019 Sep 6;8:e48627. doi: 10.7554/eLife.48627 (PMC6754230; doi:10.7554/eLife.48627)
Supplement: Supplementary file 3. — Git code repository: https://gitlab.com/csb.ethz/Kip2-SPB-Profile-Manuscript (Chen, 2019; copy archived at https://github.com/elifesciences-publications/Kip2-SPB-Profile-Manuscript). [file elife-48627-supp3.docx]

Supplementary File 3.

Stochastic simulation propensities and change vectors.

| Reaction | Propensity | Applies at | State change |
| --- | --- | --- | --- |
| Minus-end binding | $\text{free}\left(x_{1}\right)r_{\text{in}}$ | minus end site only | $x_{1}\to1$ |
| Lattice binding | $\text{free}\left(x_{i}\right)r_{\text{on}}$ | all lattice sites | $x_{i}\to1$ |
| Lattice dissociation | $\text{occ}\left(x_{i}\right)k_{\text{off}}$ | all sites except plus end | $x_{i}\to0$ |
| Stepping | $\text{occ}\left(x_{i}\right)\text{free}\left(x_{i+1}\right)k_{\text{step}}$ | all sites except plus end | $x_{i}\to0, x_{i+1}\to1$ |
| Plus-end dissociation | $\text{occ}\left(x_{N}\right)k_{\text{out}}$ | plus end site only | $x_{N}\to0$ |
